# Bis[2-(4-amino­phen­yl)-4,5-dihydro-1*H*-imidazol-3-ium] dichloride monohydrate

**DOI:** 10.1107/S1600536811050070

**Published:** 2011-11-25

**Authors:** Krešimir Molčanov, Ivana Stolić, Biserka Kojić-Prodić, Miroslav Bajić

**Affiliations:** a Laboratory of Chemical and Biological Crystallography, Department of Physical Chemistry, Rudjer Bošković Institute, POB-180, HR-10002 Zagreb, Croatia; b Department of Chemistry and Biochemistry, Faculty of Veterinary Medicine, University of Zagreb, Heinzelova 55, HR-10000 Zagreb, Croatia

## Abstract

The asymmetric unit of the title compound, 2C_9_H_12_N_3_^+^·2Cl^−^·H_2_O, comprises two mol­ecules, two chloride anions and one mol­ecule of crystal water. In the imidazolinium ring, the protonation contributes to delocalization of the positive charge over the two C—N bonds. Both chloride anions are acceptors of four hydrogen bonds in a flattened tetra­hedron environment. The donors are NH_2_ groups, the NH groups of the imidazolinium rings and the water mol­ecule. These hydrogen bonds and N—H⋯O(H_2_O) hydrogen bonds form a three-dimensional network.

## Related literature

For background and the biological activity of aromatic amidines, see: Chen *et al.* (2010 ▶); Hu *et al.* (2009 ▶); Del Poeta *et al.* (1998 ▶); Baraldi *et al.* (2004 ▶); Jarak *et al.* (2011 ▶); Neidle (2001 ▶); Stolić *et al.* (2011 ▶). For the synthesis, see Widra *et al.* (1990 ▶). For related compounds see: Jarak *et al.* (2005 ▶); Legrand *et al.* (2008 ▶). For puckering parameters, see: Cremer & Pople (1975 ▶);
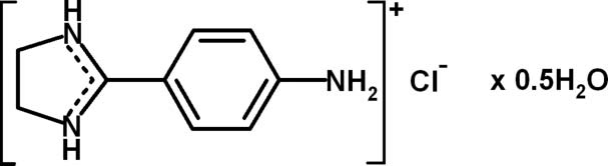


## Experimental

### 

#### Crystal data

2C_9_H_12_N_3_^+^·2Cl^−^·H_2_O
*M* *_r_* = 413.35
Orthorhombic, 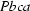

*a* = 10.5307 (2) Å
*b* = 17.9659 (4) Å
*c* = 22.4290 (5) Å
*V* = 4243.42 (16) Å^3^
*Z* = 8
Cu *K*α radiation
μ = 2.91 mm^−1^
*T* = 293 K
0.4 × 0.05 × 0.04 mm

#### Data collection

Oxford Xcalibur Nova R Ruby diffractometer
Absorption correction: multi-scan (*ABSPACK*; Oxford Diffraction, 2010 ▶) *T*_min_ = 0.389, *T*_max_ = 0.892
13695 measured reflections
4375 independent reflections
3054 reflections with *I* > 2σ(*I*)
*R*_int_ = 0.030

#### Refinement

*R*[*F* ^2^ > 2σ(*F* ^2^)] = 0.045
*wR*(*F* ^2^) = 0.116
*S* = 1.00
4375 reflections
348 parameters
3 restraints
H atoms treated by a mixture of independent and constrained refinement
Δρ_max_ = 0.24 e Å^−3^
Δρ_min_ = −0.13 e Å^−3^

### 

Data collection: *CrysAlis PRO* (Oxford Diffraction, 2010 ▶); cell refinement: *CrysAlis PRO*; data reduction: *CrysAlis PRO*; program(s) used to solve structure: *SHELXS97* (Sheldrick, 2008 ▶); program(s) used to refine structure: *SHELXL97* (Sheldrick, 2008 ▶); molecular graphics: *ORTEP-3 for Windows* (Farrugia, 1997 ▶); software used to prepare material for publication: *WinGX* (Farrugia, 1999 ▶).

## Supplementary Material

Crystal structure: contains datablock(s) global, I. DOI: 10.1107/S1600536811050070/bq2316sup1.cif

Structure factors: contains datablock(s) I. DOI: 10.1107/S1600536811050070/bq2316Isup2.hkl

Supplementary material file. DOI: 10.1107/S1600536811050070/bq2316Isup3.cml

Additional supplementary materials:  crystallographic information; 3D view; checkCIF report

## Figures and Tables

**Table 1:** Hydrogen-bond geometry (Å, °)

*D*—H⋯*A*	*D*—H	H⋯*A*	*D*⋯*A*	*D*—H⋯*A*
N1*A*—H11⋯Cl2^i^	0.86	2.45	3.296 (2)	170
N1*A*—H12⋯Cl1	0.86	2.45	3.304 (2)	170
N1*B*—H21⋯Cl2^ii^	0.86	2.59	3.448 (2)	174
N1*B*—H22⋯O1^iii^	0.86	2.02	2.882 (3)	177
N2*A*—H2*C*⋯Cl2	0.86	2.29	3.1113 (18)	160
N2*B*—H2*D*⋯Cl1^ii^	0.86	2.35	3.1615 (19)	157
N3*A*—H3*C*⋯Cl1^i^	0.86	2.36	3.1900 (17)	162
O1—H1*A*⋯Cl2^iv^	0.93 (2)	2.21 (2)	3.1329 (19)	178 (3)
O1—H1*B*⋯Cl1^v^	0.95 (2)	2.21 (2)	3.147 (2)	170 (3)

Symmetry codes: (i) 
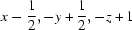
; (ii) 
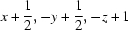
; (iii) 
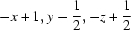
; (iv) 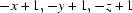; (v) 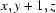.

**Fig. 1.**
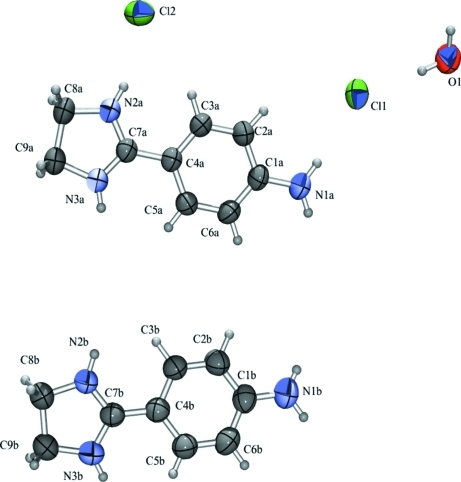
ORTEP-3 (Farrugia, 1997) drawing of the asymmetric unit of I. Displacement ellipsoids are drawn for the probability of 50% and hydrogen atoms are depicted as spheres of arbitrary radii.

**Fig. 2.**
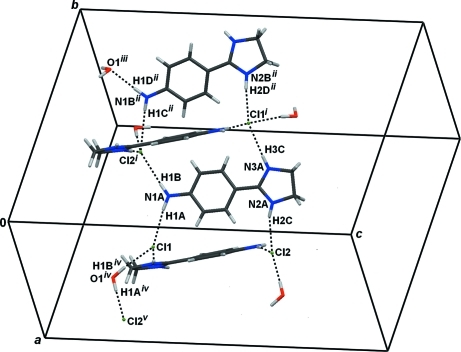
Hydrogen bonding in I. Symmetry operators: (i) x + 1/2, -y + 1/2, -z + 1; (ii) x - 1, y, z; (iii) -x + 1, y - 3/2, -z + 1/2; (iv) x, y - 1, z; (v) - x + 1, -y, -z + 1.

## supplementary crystallographic information

### Comment

Nucleic acids are important targets for many biomolecules and small molecules. Many anticancer drugs are known to exert their biological activity through bonding into minor groove of DNA. Aromatic amidines which bind strongly into the DNA minor groove exhibit outstandindg antiparasitic (Chen *et al.*, 2010), antibacterial (Hu *et al.*, 2009), antifungal (Del Poeta *et al.*, 1998), and antitumor activity (Baraldi *et al.*, 2004). The amidinium moiety is known to contribute to DNA binding of small molecules by electrostatic, van der Waals and hydrogen bonding interactions (Neidle, 2001). Aminobenzamidine derivatives are very useful building blocks for construction of target complex molecules (Jarak *et al.*, 2011). We found out that 4,5-dihydroimidazoles with cyclic amidine moiety at the terminal positions show sometimes better antitumor activity than corresponding unsubstituted or alkyl substituted amidines (Stolić *et al.*, 2011). Detail analysis of interactions of these compounds with nucleic acids can help to design more potent agents against different types of diseases.

The asymmetric unit of I comprises two molecules (labeled as A and B) and a single molecule of crystal water (Fig. 1). The five-membered rings of the cations are almost planar, the Cremer-Pople (Cremer & Pople, 1975) puckering parameters Θ being 3.2° and 0.6° for A and B molecules, respectively. The cations, however, are not planar, since mean planes of six- and five-membered rings are tilted by 9.3° and 14.8°, respectively. Both imino nitrogen atoms of the imidazolinium ring are protonated, since the imidazole is stronger proton acceptor than the amine nitrogen. The positive charge is delocalized over the two C—N bonds in the five-membered ring (Scheme 1, Fig. 1), further stabilizing the cation. The chloride anions are acceptors of four hydrogen bonds in the shapes of flattened tetrahedra with different donor groups: Cl1 accepts hydrogen bonds from two NH group of the imidazolinium ring, one NH_2_ group and a water molecule; Cl2 is surrounded by two NH_2_ groups, one imidazolinium NH and a water molecule. The molecule of crystal water is a proton donor to chloride ions and acceptor of N—H···O bonds. Thus, crystal packing comprises three-dimensional hydrogen bonding network (Fig. 2, Table 1).

### Experimental

The crude imidate ester hydrochloride (2.39 g, 12.8 mmol) prepared from 4-aminobenzonitrile (1.66 g, 14.1 mmol) in anhydrous methanol by Pinner reaction was suspended in anhydrous methanol (50 ml), 1,2-diaminoethane (12 ml) was added and mixture was refluxed for 12 h under the nitrogen atmosphere. The solvent was removed under reduced pressure and residue was recrystallized from ethanol-diethyl ether to yield 1.27 g (50.5%) of pale brown powder, m.p. 473 K; IR (ν_max_/cm^-1^): 3353, 3099, 1582, 1502, 1364, 1191, 949, 835; ^1^H NMR (DMSO-d6) δ/p.p.m.: 10.12 (s, 2H, NH), 7.76 (s, 2H, NH2), 6.65 (s, 2H, ArH), 6.46 (s, 2H, ArH), 2.50 (s, 4H, CH2).

### Refinement

The H atoms were positioned geometrically and allowed to ride on their parent atoms, with C—H = 0.93 Å and 0.97 Å for C and 0.86 Å for N atom and *U*_iso_(H) = 1.2*U*_eq_(C,N). The H atoms of water were located in difference map and then allowed to ride on their parent atoms, with O—H = 0.95 Å and 1.5*U*_eq_(O).

### Crystal data

**Table:**

2C_9_H_12_N_3_^+^·2Cl^−^·H_2_O	*F*(000) = 1744
*M**_r_* = 413.35	*D*_x_ = 1.294 Mg m^−^^3^
Orthorhombic, *P**b**c**a*	Cu *K*α radiation, λ = 1.54184 Å
Hall symbol: -P 2ac 2ab	Cell parameters from 4375 reflections
*a* = 10.5307 (2) Å	θ = 3.2–76.0°
*b* = 17.9659 (4) Å	µ = 2.91 mm^−^^1^
*c* = 22.4290 (5) Å	*T* = 293 K
*V* = 4243.42 (16) Å^3^	Prism, colourless
*Z* = 8	0.4 × 0.05 × 0.04 mm

### Data collection

**Table:**

Oxford Xcalibur Nova R Ruby diffractometer	3054 reflections with *I* > 2σ(*I*)
CCD detector, ω scans	*R*_int_ = 0.030
Absorption correction: multi-scan (ABSPACK; Oxford Diffraction, 2010)	θ_max_ = 76.2°, θ_min_ = 3.9°
*T*_min_ = 0.389, *T*_max_ = 0.892	*h* = −10→13
13695 measured reflections	*k* = −22→18
4375 independent reflections	*l* = −12→27

### Refinement

**Table:**

Refinement on *F*^2^	3 restraints
Least-squares matrix: full	H atoms treated by a mixture of independent and constrained refinement
*R*[*F*^2^ > 2σ(*F*^2^)] = 0.045	*w* = 1/[σ^2^(*F*_o_^2^) + (0.0664*P*)^2^ + 0.1666*P*] where *P* = (*F*_o_^2^ + 2*F*_c_^2^)/3
*wR*(*F*^2^) = 0.116	(Δ/σ)_max_ < 0.001
*S* = 1.00	Δρ_max_ = 0.24 e Å^−^^3^
4375 reflections	Δρ_min_ = −0.13 e Å^−^^3^
348 parameters	

### Special details

**Table:**

Geometry. All e.s.d.'s (except the e.s.d. in the dihedral angle between two l.s. planes) are estimated using the full covariance matrix. The cell e.s.d.'s are taken into account individually in the estimation of e.s.d.'s in distances, angles and torsion angles; correlations between e.s.d.'s in cell parameters are only used when they are defined by crystal symmetry. An approximate (isotropic) treatment of cell e.s.d.'s is used for estimating e.s.d.'s involving l.s. planes.

### Fractional atomic coordinates and isotropic or equivalent isotropic displacement parameters (Å^2^)

**Table:**

	*x*	*y*	*z*	*U*_iso_*/*U*_eq_	
C1A	0.15472 (18)	0.24466 (12)	0.46353 (9)	0.0591 (4)	
C2A	0.2329 (2)	0.20468 (13)	0.50240 (9)	0.0677 (5)	
H2A	0.2799	0.1647	0.488	0.081*	
C3A	0.2413 (2)	0.22367 (13)	0.56166 (9)	0.0649 (5)	
H3A	0.2947	0.1966	0.5866	0.078*	
C4A	0.17150 (17)	0.28251 (10)	0.58513 (8)	0.0529 (4)	
C5A	0.09206 (18)	0.32174 (11)	0.54635 (9)	0.0590 (4)	
H5A	0.0434	0.3609	0.561	0.071*	
C6A	0.08469 (19)	0.30346 (12)	0.48693 (9)	0.0630 (5)	
H6A	0.032	0.3309	0.4619	0.076*	
C7A	0.17897 (17)	0.30141 (10)	0.64793 (8)	0.0536 (4)	
C8A	0.2419 (2)	0.29987 (16)	0.74592 (10)	0.0797 (6)	
H811	0.3175	0.3238	0.7616	0.096*	
H812	0.2177	0.2594	0.7722	0.096*	
C9A	0.1344 (2)	0.35524 (14)	0.73860 (10)	0.0759 (6)	
H911	0.0625	0.3423	0.7636	0.091*	
H912	0.1618	0.4054	0.7481	0.091*	
N1A	0.1465 (2)	0.22627 (13)	0.40482 (8)	0.0825 (6)	
H11	0.0974	0.2511	0.3815	0.099*	
H12	0.1904	0.1898	0.391	0.099*	
N2A	0.26224 (17)	0.27362 (11)	0.68540 (8)	0.0705 (5)	
H2C	0.3218	0.2434	0.6753	0.085*	
N3A	0.10317 (17)	0.34793 (10)	0.67552 (8)	0.0671 (4)	
H3C	0.0421	0.3714	0.6584	0.081*	
C1B	0.7185 (2)	0.49540 (12)	0.38839 (10)	0.0699 (5)	
C2B	0.7994 (2)	0.48300 (13)	0.43669 (11)	0.0726 (6)	
H2B	0.8718	0.4542	0.4314	0.087*	
C3B	0.7741 (2)	0.51242 (13)	0.49165 (10)	0.0677 (5)	
H3B	0.8288	0.5024	0.5232	0.081*	
C4B	0.66745 (19)	0.55730 (11)	0.50120 (9)	0.0597 (4)	
C5B	0.5881 (2)	0.57082 (13)	0.45265 (11)	0.0679 (5)	
H5B	0.5171	0.601	0.4577	0.082*	
C6B	0.6124 (2)	0.54067 (14)	0.39764 (11)	0.0738 (6)	
H6B	0.5576	0.5505	0.3661	0.089*	
C7B	0.64277 (17)	0.58804 (11)	0.55951 (10)	0.0593 (5)	
C8B	0.6543 (2)	0.60604 (14)	0.66131 (11)	0.0752 (6)	
H821	0.7238	0.632	0.6807	0.09*	
H822	0.6143	0.5728	0.6897	0.09*	
C9B	0.5585 (2)	0.66057 (15)	0.63481 (12)	0.0809 (7)	
H921	0.4744	0.6527	0.6512	0.097*	
H922	0.5839	0.7117	0.6419	0.097*	
N1B	0.7415 (2)	0.46400 (14)	0.33459 (10)	0.0947 (7)	
H21	0.8067	0.4359	0.3298	0.114*	
H22	0.6908	0.4722	0.3053	0.114*	
N2B	0.69789 (18)	0.56590 (11)	0.60876 (8)	0.0702 (5)	
H2D	0.7541	0.5312	0.6099	0.084*	
N3B	0.56234 (18)	0.64252 (11)	0.57148 (9)	0.0762 (5)	
H3D	0.5174	0.6648	0.5449	0.091*	
Cl1	0.33702 (5)	0.08741 (3)	0.36842 (2)	0.06847 (16)	
Cl2	0.48675 (5)	0.16106 (3)	0.68365 (3)	0.08017 (19)	
O1	0.4366 (2)	0.99224 (11)	0.26032 (8)	0.0905 (5)	
H1A	0.461 (3)	0.9475 (12)	0.2776 (14)	0.136*	
H1B	0.396 (3)	1.0191 (15)	0.2913 (12)	0.136*	

### Atomic displacement parameters (Å^2^)

**Table:**

	*U*^11^	*U*^22^	*U*^33^	*U*^12^	*U*^13^	*U*^23^
C1A	0.0660 (10)	0.0683 (11)	0.0431 (10)	−0.0008 (9)	0.0021 (9)	0.0024 (9)
C2A	0.0789 (12)	0.0720 (13)	0.0522 (12)	0.0194 (10)	0.0007 (10)	−0.0040 (10)
C3A	0.0728 (11)	0.0716 (12)	0.0503 (11)	0.0160 (10)	−0.0065 (9)	−0.0001 (9)
C4A	0.0563 (9)	0.0563 (10)	0.0461 (10)	−0.0010 (8)	0.0003 (8)	0.0006 (8)
C5A	0.0666 (10)	0.0577 (10)	0.0527 (11)	0.0084 (9)	0.0027 (9)	0.0007 (8)
C6A	0.0677 (10)	0.0707 (12)	0.0506 (11)	0.0092 (10)	−0.0040 (9)	0.0071 (9)
C7A	0.0588 (9)	0.0527 (9)	0.0492 (10)	−0.0024 (8)	−0.0012 (8)	0.0008 (8)
C8A	0.0945 (15)	0.0948 (17)	0.0499 (12)	0.0214 (13)	−0.0123 (11)	−0.0105 (11)
C9A	0.0898 (14)	0.0868 (15)	0.0511 (12)	0.0187 (13)	−0.0059 (11)	−0.0109 (11)
N1A	0.1041 (14)	0.0982 (15)	0.0453 (10)	0.0272 (12)	−0.0067 (10)	−0.0056 (9)
N2A	0.0768 (10)	0.0855 (12)	0.0491 (10)	0.0234 (9)	−0.0076 (8)	−0.0075 (8)
N3A	0.0762 (10)	0.0756 (11)	0.0496 (10)	0.0201 (9)	−0.0077 (8)	−0.0088 (8)
C1B	0.0866 (13)	0.0648 (12)	0.0582 (12)	−0.0045 (11)	0.0046 (11)	0.0040 (10)
C2B	0.0807 (13)	0.0690 (13)	0.0682 (14)	0.0124 (11)	0.0058 (11)	0.0054 (11)
C3B	0.0758 (12)	0.0672 (12)	0.0601 (13)	0.0098 (10)	−0.0006 (10)	0.0069 (10)
C4B	0.0647 (10)	0.0540 (10)	0.0606 (12)	−0.0013 (9)	0.0017 (9)	0.0084 (9)
C5B	0.0686 (11)	0.0669 (12)	0.0684 (14)	0.0044 (10)	−0.0027 (10)	0.0054 (10)
C6B	0.0821 (13)	0.0794 (15)	0.0600 (13)	−0.0002 (12)	−0.0092 (11)	0.0080 (11)
C7B	0.0587 (9)	0.0559 (10)	0.0634 (12)	0.0002 (8)	0.0003 (9)	0.0057 (9)
C8B	0.0797 (13)	0.0808 (15)	0.0652 (14)	0.0153 (12)	0.0023 (11)	−0.0055 (11)
C9B	0.0854 (14)	0.0821 (15)	0.0753 (16)	0.0220 (13)	0.0018 (13)	−0.0069 (12)
N1B	0.1131 (15)	0.1059 (17)	0.0651 (13)	0.0179 (14)	−0.0007 (12)	−0.0067 (12)
N2B	0.0787 (10)	0.0727 (11)	0.0592 (10)	0.0204 (9)	−0.0019 (9)	−0.0008 (8)
N3B	0.0819 (11)	0.0759 (11)	0.0707 (12)	0.0247 (10)	−0.0048 (10)	0.0015 (9)
Cl1	0.0833 (3)	0.0644 (3)	0.0577 (3)	−0.0134 (2)	0.0066 (2)	−0.0012 (2)
Cl2	0.0760 (3)	0.0766 (3)	0.0879 (4)	0.0119 (3)	0.0061 (3)	0.0194 (3)
O1	0.1249 (14)	0.0828 (11)	0.0637 (10)	0.0098 (11)	0.0000 (10)	0.0030 (8)

### Geometric parameters (Å, °)

**Table:**

C1A—N1A	1.360 (3)	C1B—C2B	1.396 (3)
C1A—C6A	1.391 (3)	C1B—C6B	1.397 (3)
C1A—C2A	1.398 (3)	C2B—C3B	1.367 (3)
C2A—C3A	1.375 (3)	C2B—H2B	0.93
C2A—H2A	0.93	C3B—C4B	1.399 (3)
C3A—C4A	1.391 (3)	C3B—H3B	0.93
C3A—H3A	0.93	C4B—C5B	1.394 (3)
C4A—C5A	1.398 (3)	C4B—C7B	1.443 (3)
C4A—C7A	1.451 (3)	C5B—C6B	1.372 (3)
C5A—C6A	1.375 (3)	C5B—H5B	0.93
C5A—H5A	0.93	C6B—H6B	0.93
C6A—H6A	0.93	C7B—N2B	1.310 (3)
C7A—N3A	1.311 (2)	C7B—N3B	1.322 (3)
C7A—N2A	1.313 (2)	C8B—N2B	1.456 (3)
C8A—N2A	1.453 (3)	C8B—C9B	1.527 (3)
C8A—C9A	1.516 (3)	C8B—H821	0.97
C8A—H811	0.97	C8B—H822	0.97
C8A—H812	0.97	C9B—N3B	1.458 (3)
C9A—N3A	1.458 (3)	C9B—H921	0.97
C9A—H911	0.97	C9B—H922	0.97
C9A—H912	0.97	N1B—H21	0.86
N1A—H11	0.86	N1B—H22	0.86
N1A—H12	0.86	N2B—H2D	0.86
N2A—H2C	0.86	N3B—H3D	0.86
N3A—H3C	0.86	O1—H1A	0.928 (17)
C1B—N1B	1.354 (3)	O1—H1B	0.947 (17)
			
N1A—C1A—C6A	121.06 (19)	N1B—C1B—C6B	121.2 (2)
N1A—C1A—C2A	121.1 (2)	C2B—C1B—C6B	117.7 (2)
C6A—C1A—C2A	117.84 (19)	C3B—C2B—C1B	121.3 (2)
C3A—C2A—C1A	120.9 (2)	C3B—C2B—H2B	119.4
C3A—C2A—H2A	119.6	C1B—C2B—H2B	119.4
C1A—C2A—H2A	119.6	C2B—C3B—C4B	121.1 (2)
C2A—C3A—C4A	121.37 (19)	C2B—C3B—H3B	119.4
C2A—C3A—H3A	119.3	C4B—C3B—H3B	119.4
C4A—C3A—H3A	119.3	C5B—C4B—C3B	117.5 (2)
C3A—C4A—C5A	117.64 (18)	C5B—C4B—C7B	122.24 (19)
C3A—C4A—C7A	121.11 (17)	C3B—C4B—C7B	120.26 (19)
C5A—C4A—C7A	121.23 (17)	C6B—C5B—C4B	121.5 (2)
C6A—C5A—C4A	121.10 (19)	C6B—C5B—H5B	119.3
C6A—C5A—H5A	119.4	C4B—C5B—H5B	119.3
C4A—C5A—H5A	119.4	C5B—C6B—C1B	120.8 (2)
C5A—C6A—C1A	121.15 (19)	C5B—C6B—H6B	119.6
C5A—C6A—H6A	119.4	C1B—C6B—H6B	119.6
C1A—C6A—H6A	119.4	N2B—C7B—N3B	109.7 (2)
N3A—C7A—N2A	110.29 (18)	N2B—C7B—C4B	124.63 (18)
N3A—C7A—C4A	125.06 (17)	N3B—C7B—C4B	125.64 (19)
N2A—C7A—C4A	124.64 (18)	N2B—C8B—C9B	102.17 (19)
N2A—C8A—C9A	102.81 (17)	N2B—C8B—H821	111.3
N2A—C8A—H811	111.2	C9B—C8B—H821	111.3
C9A—C8A—H811	111.2	N2B—C8B—H822	111.3
N2A—C8A—H812	111.2	C9B—C8B—H822	111.3
C9A—C8A—H812	111.2	H821—C8B—H822	109.2
H811—C8A—H812	109.1	N3B—C9B—C8B	102.61 (18)
N3A—C9A—C8A	102.38 (17)	N3B—C9B—H921	111.2
N3A—C9A—H911	111.3	C8B—C9B—H921	111.2
C8A—C9A—H911	111.3	N3B—C9B—H922	111.2
N3A—C9A—H912	111.3	C8B—C9B—H922	111.2
C8A—C9A—H912	111.3	H921—C9B—H922	109.2
H911—C9A—H912	109.2	C1B—N1B—H21	120
C1A—N1A—H11	120	C1B—N1B—H22	120
C1A—N1A—H12	120	H21—N1B—H22	120
H11—N1A—H12	120	C7B—N2B—C8B	113.13 (18)
C7A—N2A—C8A	112.10 (18)	C7B—N2B—H2D	123.4
C7A—N2A—H2C	124	C8B—N2B—H2D	123.4
C8A—N2A—H2C	124	C7B—N3B—C9B	112.36 (19)
C7A—N3A—C9A	112.22 (17)	C7B—N3B—H3D	123.8
C7A—N3A—H3C	123.9	C9B—N3B—H3D	123.8
C9A—N3A—H3C	123.9	H1A—O1—H1B	105 (2)
N1B—C1B—C2B	121.1 (2)		
			
N1A—C1A—C2A—C3A	179.7 (2)	N1B—C1B—C2B—C3B	−177.6 (2)
C6A—C1A—C2A—C3A	−0.8 (3)	C6B—C1B—C2B—C3B	1.7 (4)
C1A—C2A—C3A—C4A	0.7 (4)	C1B—C2B—C3B—C4B	−1.3 (4)
C2A—C3A—C4A—C5A	0.1 (3)	C2B—C3B—C4B—C5B	0.0 (3)
C2A—C3A—C4A—C7A	178.9 (2)	C2B—C3B—C4B—C7B	−179.8 (2)
C3A—C4A—C5A—C6A	−0.9 (3)	C3B—C4B—C5B—C6B	0.8 (3)
C7A—C4A—C5A—C6A	−179.66 (19)	C7B—C4B—C5B—C6B	−179.5 (2)
C4A—C5A—C6A—C1A	0.9 (3)	C4B—C5B—C6B—C1B	−0.3 (4)
N1A—C1A—C6A—C5A	179.4 (2)	N1B—C1B—C6B—C5B	178.4 (2)
C2A—C1A—C6A—C5A	0.0 (3)	C2B—C1B—C6B—C5B	−0.9 (3)
C3A—C4A—C7A—N3A	−169.1 (2)	C5B—C4B—C7B—N2B	165.7 (2)
C5A—C4A—C7A—N3A	9.6 (3)	C3B—C4B—C7B—N2B	−14.6 (3)
C3A—C4A—C7A—N2A	10.2 (3)	C5B—C4B—C7B—N3B	−14.6 (3)
C5A—C4A—C7A—N2A	−171.1 (2)	C3B—C4B—C7B—N3B	165.2 (2)
N2A—C8A—C9A—N3A	4.1 (3)	N2B—C8B—C9B—N3B	0.3 (3)
N3A—C7A—N2A—C8A	2.6 (3)	N3B—C7B—N2B—C8B	0.8 (3)
C4A—C7A—N2A—C8A	−176.8 (2)	C4B—C7B—N2B—C8B	−179.5 (2)
C9A—C8A—N2A—C7A	−4.3 (3)	C9B—C8B—N2B—C7B	−0.7 (3)
N2A—C7A—N3A—C9A	0.5 (3)	N2B—C7B—N3B—C9B	−0.6 (3)
C4A—C7A—N3A—C9A	179.9 (2)	C4B—C7B—N3B—C9B	179.7 (2)
C8A—C9A—N3A—C7A	−3.1 (3)	C8B—C9B—N3B—C7B	0.1 (3)

### Hydrogen-bond geometry (Å, °)

**Table:**

*D*—H···*A*	*D*—H	H···*A*	*D*···*A*	*D*—H···*A*
N1A—H11···Cl2^i^	0.86	2.45	3.296 (2)	170.
N1A—H12···Cl1	0.86	2.45	3.304 (2)	170.
N1B—H21···Cl2^ii^	0.86	2.59	3.448 (2)	174.
N1B—H22···O1^iii^	0.86	2.02	2.882 (3)	177.
N2A—H2C···Cl2	0.86	2.29	3.1113 (18)	160
N2B—H2D···Cl1^ii^	0.86	2.35	3.1615 (19)	157.
N3A—H3C···Cl1^i^	0.86	2.36	3.1900 (17)	162.
O1—H1A···Cl2^iv^	0.93 (2)	2.21 (2)	3.1329 (19)	178 (3)
O1—H1B···Cl1^v^	0.95 (2)	2.21 (2)	3.147 (2)	170 (3)

Symmetry codes: (i) *x*−1/2, −*y*+1/2, −*z*+1; (ii) *x*+1/2, −*y*+1/2, −*z*+1; (iii) −*x*+1, *y*−1/2, −*z*+1/2; (iv) −*x*+1, −*y*+1, −*z*+1; (v) *x*, *y*+1, *z*.
